# Intolerance of uncertainty, paranoia, and prodromal symptoms: Comparisons between a schizophrenia spectrum disorder, anxiety disorder and non‐clinical sample

**DOI:** 10.1111/papt.12599

**Published:** 2025-06-02

**Authors:** Jayne Morriss, Lyn Ellett

**Affiliations:** ^1^ School of Psychology, Faculty of Environmental and Life Sciences University of Southampton Southampton UK

**Keywords:** anxiety, intolerance of uncertainty, paranoia, prodromal symptoms of schizophrenia, schizophrenia

## Abstract

**Background:**

Greater Intolerance of Uncertainty (IU: the tendency to find uncertainty negative) is associated with greater paranoia (mistrust of others) in clinical samples with schizophrenia spectrum disorders (SSDs). Questions remain on whether the relationship between IU and paranoia/prodromal symptoms is: (1) specific over other related negative affective traits and cognitive biases, and (2) specific to SSDs or is transdiagnostic.

**Methods:**

To examine these research questions, we conducted a survey in those with SSDs (*n* = 103), anxiety disorders (*n* = 102) a non‐clinical sample (*n* = 102). Questionnaires included: IU, paranoia, prodromal symptoms of schizophrenia, neuroticism and jumping to conclusions bias.

**Results:**

IU, neuroticism and jumping to conclusions bias were elevated in those with SSDs and anxiety disorders, compared to the non‐clinical group. Both paranoia and prodromal symptoms were highest in those with SSDs, then anxiety disorders and lowest in the non‐clinical group. Greater IU was associated with greater paranoia and prodromal symptoms across SSDs, anxiety disorders and a non‐clinical sample. The majority of the relationships between IU and paranoia/prodromal symptoms remained significant when controlling for neuroticism and the jumping to conclusions bias. However, the relationship between IU and paranoia in the SSD group was not specific over the jumping to conclusions bias.

**Discussion:**

These findings highlight the potentially transdiagnostic role of IU in paranoia/prodromal symptoms across SSDs and anxiety disorders, which has implications for the development of transdiagnostic treatment interventions for SSDs and anxiety disorders.

Intolerance of Uncertainty (IU) refers to the tendency to find uncertainty negative (Carleton, 2016a, 2016b; Freeston et al., 1994). A wealth of research has shown that individuals with higher levels of IU perceive uncertain situations as more threatening and unsafe (Cupid et al., 2021; Pepperdine et al., 2018), and under such conditions, they tend to experience heightened negative emotions (Morriss, Goh, et al., 2023) and increased physiological arousal (Morriss, Abend, et al., 2023; Tanovic et al., 2018). IU is normally distributed across community samples (Carleton et al., 2012; Hong & Lee, 2015). Moreover, IU is a transdiagnostic dimension, as the IU total score is higher across a variety of mental health conditions, including anxiety, mood, eating and schizophrenia spectrum disorders (McEvoy et al., 2019; Morriss, Butler, & Ellett, 2024; Morriss, Gaudiano, et al., 2024).

There is growing evidence that IU can be modified through gold‐standard treatments, including transdiagnostic treatments for anxiety‐related disorders (for meta‐analysis, see Miller & McGuire, 2023). Based on these advancements, the examination of IU from both basic and clinical research perspectives has become popular over the last decade (Morriss, Abend, et al., 2023; Shihata et al., 2016; Tanovic et al., 2018). The focus of this research has been to identify how IU contributes to anxiety (e.g. physiological arousal, worry) and depression (e.g. anhedonia, rumination) symptoms within community samples (e.g. Hong & Lee, 2015) and those with anxiety and mood‐related disorders (e.g. Mahoney & McEvoy, 2012). Furthermore, research has begun to investigate how therapeutic principles can be leveraged to target IU and associated symptoms such as anxiety (e.g. Li et al., 2021).

A recent emerging trend is to examine how IU modulates key symptoms within schizophrenia spectrum disorders (SSDs) such as delusions and paranoia (Bredemeier et al., 2019; Lebert et al., 2021; White & Gumley, 2010). A systematic review of 10 studies within community, at‐risk and SSD samples highlighted how IU is positively associated with delusions and paranoia symptoms (e.g. beliefs that others are intending to harm, mistrust of others) (Morriss, Butler, & Ellett, 2024). As far as we are aware, only a few studies to date have examined the specificity of the relationship between IU and paranoia symptoms, over other broader negative affective traits (Lopes & Jaspal, 2025; Morriss, Gaudiano, et al., 2024). In an international community sample across five sites (*n* = 2510), it was observed that IU was positively associated with paranoia, even when controlling for negative beliefs about the self and others. Despite this progress, there remain questions about the specificity of the relationship between IU and paranoia. First, it is yet to be established whether the relationship between IU and paranoia is specific over other transdiagnostic higher‐order negative affective traits such as neuroticism, and lower‐order cognitive biases such as jumping to conclusions, both of which have been linked to paranoia symptoms in the general population (Barrantes‐Vidal et al., 2009; Freeman et al., 2008) and those with SSDs (Freeman et al., 2013; Krabbendam et al., 2002). Second, it is unknown whether the relationship between IU and paranoia is transdiagnostic across SSDs and highly comorbid mental health conditions such as anxiety disorders (for review of comorbidity see, Braga et al., 2013), or whether the strength of the relationship between IU and paranoia is stronger in SSDs, compared to anxiety disorders. Addressing these questions will clarify whether IU and paranoia interactions are unique, over other related and overlapping transdiagnostic dimensions. Such findings will inform us about the relevance and benefit of IU as a potential treatment target for alleviating paranoia symptoms in SSDs and/or anxiety disorders.

Prodromal symptoms related to schizophrenia consist of changes in emotional experiences, perceptual abnormalities and unusual thought content. Prodromal symptoms in schizophrenia have been shown to predict psychotic episodes within 6–12 months (Yung et al., 1998, 2003). Thus, prodromal symptoms may be useful for identifying who may be at risk of experiencing psychotic episodes, and how psychotic episodes may develop in SSDs (Hall, 2018). Notably, both anxious and paranoid states are common prodromal symptoms in schizophrenia (for review see, Yung & McGorry, 1996). Given that higher IU is associated with both greater anxiety and paranoia symptoms in community samples (Morriss, Butler, & Ellett, 2024; Morriss, Gaudiano, et al., 2024) and clinical samples with SSDs (for review see, Morriss, Butler, & Ellett, 2024), it is possible that IU may represent a risk factor for prodromal symptoms as well. Indeed, one study has shown that IU is elevated in those with an at‐risk state of psychosis, compared to controls (Broome et al., 2007). It is important to examine whether this effect can be replicated in larger non‐clinical samples and clinical samples with anxiety disorders and/or SSDs, in order to address whether higher IU is associated with greater prodromal symptoms in general and whether this relationship becomes stronger based on disorder status, for example, higher IU is associated with greater prodromal symptoms in SSDs, versus anxiety disorders and non‐clinical samples. Examining this relationship will provide insights into the robustness of the relationship between IU and prodromal symptoms, which will have implications for potential avenues for preventive medicine. For instance, targeting IU at the earlier stages of the prodrome may prevent the development of psychotic episodes (for discussion about prevention see Hall, 2018).

The following study aimed to examine:

1. The extent to which IU, paranoia, prodromal symptoms, neuroticism and jumping to conclusions bias vary by disorder group status, that is, SSD, anxiety disorder and non‐clinical sample.
2. Whether the relationship between IU and paranoia/prodromal symptoms is specific over the higher‐order broader negative affective trait, neuroticism (John, 2021) and the lower‐order cognitive bias, jumping to conclusions (Peters et al., 2014) within those with SSDs, anxiety disorders and without any mental health conditions.
3. Whether the relationship between IU and paranoia/prodromal symptoms of schizophrenia is unique to SSDs or transdiagnostic across SSDs, anxiety disorders and those without any mental health conditions.

To address these research questions, we conducted a survey on a sample of individuals with SSDs (*n* = 103), anxiety disorders (*n* = 102) and a non‐clinical sample (*n* = 102). We measured IU, paranoia, prodromal symptoms of schizophrenia, neuroticism and jumping to conclusions bias via questionnaires. We tested the following hypotheses:

1. Higher IU, neuroticism and jumping to conclusions bias would be observed in the SSD and anxiety disorder groups, compared to a non‐clinical sample. We based these hypotheses on prior research (Barrantes‐Vidal et al., 2009; Freeman et al., 2013; Morriss, Butler, & Ellett, 2024; So et al., 2016). However, as far as we are aware there have not been any direct comparisons between those with SSDs and anxiety disorders for IU, neuroticism and jumping to conclusions bias.
2. Paranoia symptoms and prodromal symptoms would be highest in SSD, then anxiety disorders, and then a non‐clinical sample. We based these hypotheses on a wealth of research that have demonstrated paranoia (e.g. Freeman, 2016) and prodromal symptoms of schizophrenia (Yung et al., 1998, 2003) to be central to SSDs. However, both paranoia (Reich & Braginsky, 1994; Taylor & Stopa, 2013) and prodromal symptoms (Rietdijk et al., 2011; Shioiri et al., 2007) can occur in those with anxiety disorders as well.
3. Higher IU may be associated with higher paranoia/prodromal symptoms, over neuroticism and jumping to conclusions bias. This hypothesis was based on a previous study suggesting specificity for IU in relation to paranoia symptoms (Morriss, Butler, & Ellett, 2024; Morriss, Gaudiano, et al., 2024).
4. The strength of the relationships between IU and paranoia/prodromal symptoms may be more pronounced in SSDs, compared to anxiety disorders and a non‐clinical sample. Alternatively, the relationship between IU and paranoia/prodromal symptoms may be similar across the different groups, suggesting it to be transdiagnostic. This exploratory hypothesis reflects the lack of prior research findings on this topic.

## METHOD

### Participants

Participants took part in the study if they had a primary diagnosis of a schizophrenia spectrum disorder (including psychosis symptoms related to a bipolar disorder, personality disorder or post‐traumatic stress disorder) (*n* = 103), an anxiety disorder (*n* = 102) or no existing or past diagnoses of mental health conditions (*n* = 102). Participants were assigned to the SSD and anxiety disorder groups based on their primary diagnosis but could have secondary diagnoses of other disorders (see Table 1).

**TABLE 1:** Demographic information per group.

	Schizophrenia spectrum disorders (*n* = 103)	Anxiety disorders (*n* = 102)	Non‐clinical sample (*n* = 102)
Age in years (SD)	33.04 (10.57)	42.17 (11.37)	41.06 (13.34)
Country of residence (%)			
UK	44.66	100.00	100.00
USA	55.33	0.00	0.00
Education (%)			
Primary	1.00	0.00	0.00
Partial secondary	1.90	1.00	1.00
Secondary	33.00	10.80	15.70
Post secondary	17.50	28.40	17.60
University undergraduate	35.00	41.20	49.00
University postgraduate	9.70	18.60	16.70
Prefer not to say	1.90	0.00	0.00
Employment status (%)			
Full time	34.00	52.00	61.80
Part time	24.30	19.60	15.70
Retired	3.90	7.80	10.80
Student	5.80	2.90	4.90
Other	28.20	17.60	5.90
Prefer not to say	3.90	0.00	1.00
Ethnicity (%)			
Black	7.80	0.00	6.90
East Asian	1.90	1.00	2.90
Hispanic	8.70	0.00	1.00
Subcontinental Asian	1.00	2.90	4.90
White	75.70	92.20	80.00
Other	4.90	3.90	3.90
Prefer not to say	0.00	0.00	2.00
Gender (%)			
Female	44.70	79.40	47.10
Male	44.70	20.60	52.90
Non binary	5.80	0.00	0.00
Transgender	2.90	0.00	0.00
Use another term to describe gender	1.90	0.00	0.00
Self‐reported mental health conditions (%)			
Anxiety disorder (general)	83.49	94.11	0.00
Bipolar disorder	24.27	0.00	0.00
Major depressive disorder	77.66	50.98	0.00
Obsessive‐compulsive disorder	23.30	5.88	0.00
Panic disorder	22.33	10.78	0.00
Personality disorder	10.67	0.00	0.00
Post‐traumatic stress disorder	45.63	7.84	0.00
Psychosis (including Schizophrenia)	73.78	1.96	0.00
Specific phobia	8.73	6.86	0.00

Participants were recruited via the Prolific platform. For demographic characteristics of each group, see Table 1. Participants self‐reported their mental health diagnoses, who had diagnosed them (general practitioner *n* = 90; psychiatrist *n* = 81; psychologist *n* = 15; themselves *n* = 8; other mental health staff *n* = 2; unspecified *n* = 9), and whether they currently receive support from mental health services (yes *n* = 78; no *n* = 119; unspecified *n* = 9).

Our sample size was based on the following power analyses. First, we conducted a power analysis for a one‐way ANOVA to assess group differences between those diagnosed with SSDs, Anxiety Disorders and a non‐clinical control: effect size *f* = 0.25, *α* = 0.05, 1 *β* err prob. = 0.8, number of groups = 3. The power analysis suggested a total sample size of 159 (53 per group). Second, we conducted a power analysis for correlational tests between measures: tails = 2, effect size = 0.3, *α* = 0.05, 1 *β* err prob. = 0.8. This power analysis suggested a total sample size of 82. The medium effect sizes used for both power analyses were based on a prior systematic review on the relationship between IU and paranoia (Morriss, Butler, & Ellett, 2024; Morriss, Gaudiano, et al., 2024). We oversampled to increase statistical power.

Ethical approval was obtained from the University of Southampton Ethics Committee (ERGO: 92265) in the UK.

### Questionnaires

*The Intolerance of Uncertainty Scale – 12 items* (IU, Carleton et al., 2007) consists of 12‐items which are rated on a 5‐point scale of *1 ‘not characteristic of me’* to 5 *‘very characteristic of me’* (range 12–60). In the current sample, the Cronbach's alpha was excellent for total IU, α = .93.

*The Revised Green* et al., *Paranoid Thoughts Scale* (RGPTS, Freeman et al., 2021) is an 18‐item measure comprised of two subscales: ideas of reference (8 items) and persecution (10 items). Items are rated on a 5‐point scale of *0–not at all* to *4–totally* and exhibit reliability across the paranoia continuum. In the current study, because we were interested in paranoia specifically (as opposed to ideas of reference), we used the persecution subscale only (range 0–40), and the Cronbach's alpha was excellent, α = .95.

*The Prodromal Questionnaire* (PQ‐16, Ising et al., 2012) is a 16‐item measure that examines prodromal symptoms of psychosis. Items are rated on a binary scale of true and false (range 0–16). If an item is rated true, then an additional rating of distress is provided from 0 ‘no’ to 3 ‘severe’ (range 0–48). For this study, we used the standard scoring system based on the items for the binary scale. A cutoff of 6 or more symptoms is associated with high specificity (87%) and sensitivity (87%) of identifying ultra high‐risk for developing psychosis (Ising et al., 2012). The Cronbach's alpha was good, α = .88.

*The Big Five Inventory—10 items* (BFI‐10, Rammstedt & John, 2007) is a shortened scale that measures extraversion (2 items), agreeableness (2 items), conscientiousness (2 items), neuroticism (2 items) and openness (2 items). Items are rated on a 5‐point scale from 1 ‘*disagree strongly’* to 5 *‘agree strongly’*. In this study, we used the neuroticism subscale only (range 1–10), and the Cronbach's alpha was good, α = .82.

*The Cognitive Biases Questionnaire for psychosis* (CBQp, Peters et al., 2014) is a 30‐item measure comprised of five subscales: jumping to conclusions (6 items), intentionalising (6 items), catastrophising (6 items), emotional reasoning (6 items) and dichotomous thinking (6 items). The questionnaire presents everyday situations with three response choices. Answers with an absence of bias equal one point, those with some bias equal two points and those with a greater bias equal three points. In the current study, we used the jumping to conclusions bias subscale only (range 0–15), and the Cronbach's alpha was α = .68.

### Procedure

Participants initiated the study and consented to take part via the Prolific platform. Participants clicked a link and were directed to the Qualtrics survey platform to complete the questionnaires. To prevent missing data, participants were reminded to respond to all questions on each page before progressing through the survey. To ensure the accuracy of the data, completion time was monitored with an a priori criterion that any participant taking less than half of the median completion time would be excluded (none were excluded on this basis). Participants were paid for their time (£9/h) directly through the Prolific platform.

### Data analysis plan

All statistical analyses were conducted in SPSS 29.0 (SPSS, Inc.; Chicago, Illinois).

To check for outliers and that the data met assumptions of normality, we assessed the descriptive statistics (i.e. skewness and kurtosis values) and histograms of the dependent variables per group (based on recommendations by Byrne, 2010). First, one‐way ANOVAs with group as a factor (SSD, anxiety disorder, non‐clinical sample) were conducted to address whether IU, paranoia, prodromal symptoms, neuroticism and jumping to conclusion bias varied by group. Second, correlations were conducted for each group separately to examine whether there were statistically significant associations between the variables (e.g. IU, paranoia, prodromal symptoms, neuroticism, jumping to conclusions bias). Third, partial correlations were conducted for each group separately to examine whether associations between IU and paranoia/prodromal symptoms were specific, over and above neuroticism and the jumping to conclusions bias. Fourth, the significance of the difference between the partial correlation coefficients for each group was examined to test whether associations between IU and paranoia/prodromal symptoms were unique for each group.

Correction for multiple comparisons was conducted using the Benjamini‐Hochberg method. The corrections were applied separately to each set of tests (follow up pairwise comparisons from the ANOVAs, correlations, partial correlations).

## RESULTS

All of the dependent variables per group were normally distributed. Table 2 shows the descriptive statistics per group. Table 3 summarises the correlations between all variables per group. Table 4 presents the partial correlations between IU and paranoia/prodromal symptoms per group. All significant statistics reported survived corrections for multiple comparisons: adjusted value for follow up pairwise comparisons for the ANOVAs *p* < .0225; adjusted value for the correlations *p* < .015; adjusted value for the partial correlations (*p* < .045).

**TABLE 2:** Summary of means (SD) for each questionnaire per group.

	Schizophrenia spectrum disorders (*n* = 103)	Anxiety disorders (*n* = 102)	Non‐clinical sample (*n* = 102)
Intolerance of uncertainty	41.16 (11.53)	39.62 (10.33)	32.37 (9.02)
Jumping to conclusions bias	11.87 (2.67)	11.61 (2.16)	9.69 (2.01)
Neuroticism	7.78 (2.39)	7.84 (2.01)	5.28 (2.18)
Paranoia	15.88 (12.53)	6.88 (9.19)	3.70 (5.67)
Prodromal symptoms	10.12 (4.20)	4.97 (3.06)	2.40 (2.23)

*Note*: constructs were measured using IU‐12 (Intolerance of Uncertainty), RGPTS (Paranoia), PQ‐16 (Prodromal Symptoms), BFI‐10 (Neuroticism), CBQp (Jumping to Conclusions Bias).

**TABLE 3:** Summary of correlations per group.

	Schizophrenia spectrum disorders (*n* = 103)					Anxiety disorders (*n* = 102)					Non‐clinical sample (*n* = 102)				
	1	2	3	4	5	1	2	3	4	5	1	2	3	4	5
1. Intolerance of uncertainty	–					–					–				
2. Jumping to conclusions bias	.51**	–				.41**	–				.40**	–			
3. Neuroticism	.68**	.43**	–			.46**	.24*	–			.68**	.44**	–		
4. Paranoia	.36**	.52**	.24*	–		.41**	.23*	.32**	–		.36**	.28*	.24*	–	
5. Prodromal symptoms	.38**	.45**	.35**	.49**	–	.46**	.34**	.27*	.33**	–	.41**	.34**	.35**	.41**	–

*Note*: constructs were measured using IU‐12 (Intolerance of Uncertainty), RGPTS (Paranoia), PQ‐16 (Prodromal Symptoms), BFI‐10 (Neuroticism), CBQp (Jumping to Conclusions Bias). **p < .001, *p < .05.

**TABLE 4:** Summary of partial correlations per group.

	Schizophrenia spectrum disorders (*n* = 103)	Anxiety disorders (*n* = 102)	Non‐clinical sample (*n* = 102)
*Partial correlation for Intolerance of Uncertainty, controlling for Neuroticism*			
1. Paranoia	.28*	.31*	.28*
2. Prodromal symptoms	.21*	.39**	.30*
*Partial correlation for intolerance of uncertainty, controlling for jumping to conclusions bias*			
1. Paranoia	.13	.35**	.28*
2. Prodromal symptoms	.20*	.37**	.32**

*Note*: Constructs were measured using IU‐12 (Intolerance of Uncertainty), RGPTS (Paranoia), PQ‐16 (Prodromal Symptoms), BFI‐10 (Neuroticism), CBQp (Jumping to Conclusions Bias). **p < .001, *p < .05.

### Group differences

As expected, there were significant differences between groups based on IU [*F*(2,304) = 21.050, *p* < .001; see Table 2]. Scores for IU were elevated in those with SSDs and anxiety disorders, compared to the non‐clinical group, *p*s < 001. Scores for IU did not significantly differ for those with SSDs and anxiety disorders, *p* = .288.

Furthermore, there were significant differences between groups on paranoia [*F*(2,304) = 44.763, *p* < .001; see Table 2] and prodromal symptoms [*F*(2,304) = 147.709, *p* < .001; see Table 2]. Both paranoia and prodromal symptoms were highest in those with SSDs, then anxiety disorders and lowest in the non‐clinical group, *p*s < .05.

There were significant differences between groups for neuroticism [*F*(2,304) = 44.939, *p* < .001; see Table 2] and jumping to conclusions bias [*F*(2,304) = 27.297, *p* < .001; see Table 2]. Scores for neuroticism and jumping to conclusions bias were elevated in those with SSDs and anxiety disorders, compared to the non‐clinical group, *p*s < 001. Scores for neuroticism and jumping to conclusions bias did not significantly differ for those with SSDs and anxiety disorders, *ps* > .4.

### Correlations

**FIGURE 1:**
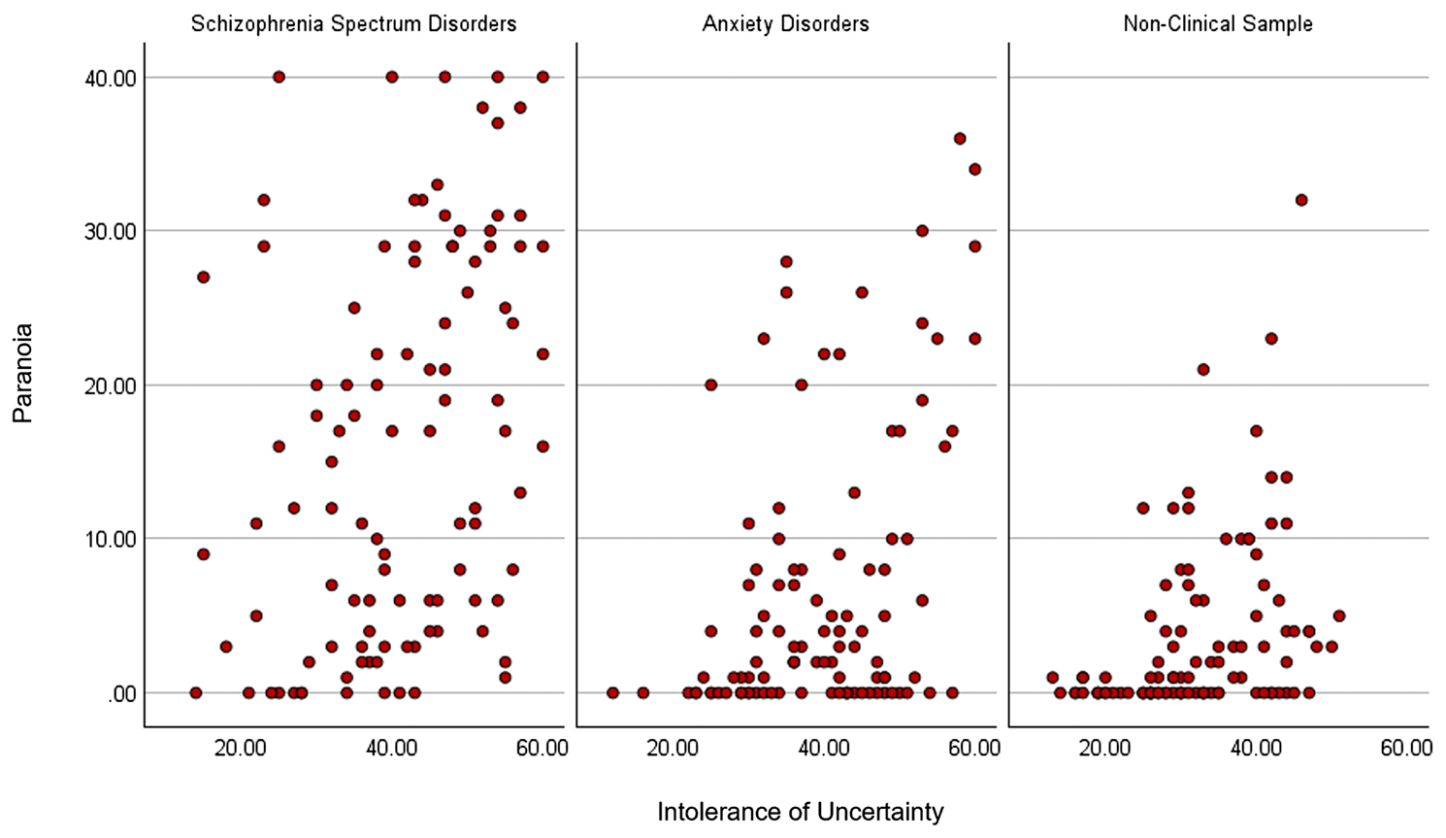
Greater intolerance of uncertainty is associated with greater paranoia symptoms across schizophrenia spectrum disorders, anxiety disorders and a non‐clinical sample.

**FIGURE 2:**
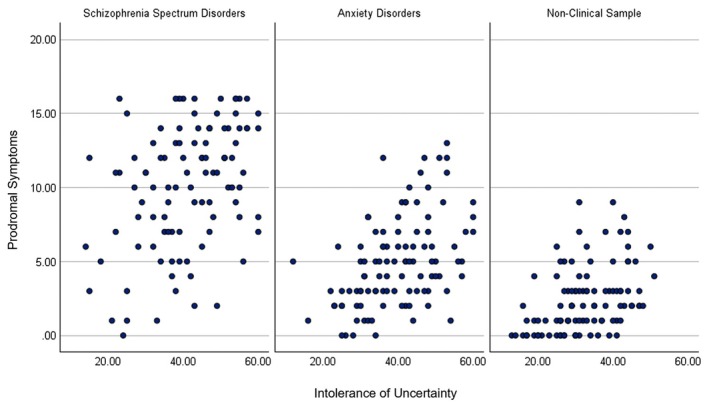
Greater intolerance of uncertainty is associated with greater prodromal symptoms across schizophrenia spectrum disorders, anxiety disorders and a non‐clinical sample.

The correlational results show that greater IU is associated with greater paranoia and prodromal symptoms across the SSDs, anxiety disorders and non‐clinical groups, *r*'s .36–46, *p*s < .001 (see Table 3 and Figures 1 and 2).

Similar relationships between neuroticism/jumping to conclusions bias with paranoia and prodromal symptoms were observed across SSDs, anxiety disorders and non‐clinical groups, *r*'s .23–52, *p*s < .05 (see Table 3).

IU, neuroticism and jumping to conclusions bias were all significantly positively correlated, *r*'s .24–68, *p*s < .05 (see Table 3).

### Partial correlations

The relationship between IU and paranoia across SSDs, anxiety disorders and the non‐clinical sample remained significant when controlling for neuroticism, *r*'s .28–31, *p*s < .05 (see Table 4). However, the relationship between IU and paranoia was no longer significant for SSD group when controlling for the jumping to conclusions bias *r =* .13, *p* < .05 (see Table 4). Although, the relationship between IU and paranoia across anxiety disorders and the non‐clinical sample, remained significant when controlling for the jumping to conclusion bias, *r*'s .28–35, *p*s < .05 (see Table 4).

Furthermore, the relationship between IU and prodromal symptoms across SSDs, anxiety disorders and the non‐clinical sample remained significant when controlling for neuroticism and jumping to conclusions bias, *r*'s .20–39, *p*s < .05 (see Table 4).

### Significance of the difference between the partial correlations

There were no significant differences between the partial correlation coefficients for IU and paranoia/prodromal symptoms across SSDs, anxiety disorders and the non‐clinical sample, *p*s > .09.

## DISCUSSION

In the present study, we examined the relationships among IU, paranoia, prodromal symptoms of schizophrenia, neuroticism and jumping to conclusions bias in those with SSDs, anxiety disorders and a non‐clinical sample. Our findings reveal that IU, neuroticism and the jumping to conclusions bias were all elevated in individuals with SSDs and anxiety disorders compared to the non‐clinical sample. Paranoia and prodromal symptoms were most pronounced in those with SSDs, followed by those with an anxiety disorder and were least evident in the non‐clinical sample. Correlational findings showed that greater IU was associated with greater paranoia and prodromal symptoms across SSDs, anxiety disorders and a non‐clinical sample. Most of the relationships between IU and paranoia/prodromal symptoms remained significant when controlling for neuroticism and the jumping to conclusions bias. However, the relationship between IU and paranoia in the SSD group was not specific over the jumping to conclusions bias. These findings suggest that IU may play a transdiagnostic role in paranoia and prodromal symptoms across SSDs and anxiety disorders. This has implications for developing treatment interventions that can target IU as a common factor in both conditions.

The findings provide evidence that high levels of IU are observed in both SSDs and anxiety disorders, compared to non‐clinical controls. While elevated IU has been observed separately in SSDs (Morriss, Butler, & Ellett, 2024) and anxiety disorders (Carleton et al., 2012), to our knowledge, this study is the first to directly compare IU levels across these two populations. Furthermore, both neuroticism and jumping to conclusions bias scores were similar in the SSD and anxiety disorder groups. The comparable levels of neuroticism for both SSD and anxiety disorders are unsurprising, given that there is substantial evidence for higher neuroticism in SSDs (for review see, Berenbaum & Fujita, 1994) and anxiety disorders (for review see, Clark et al., 1994). The pattern of results for the jumping to conclusions bias is in line with research in SSDs (So et al., 2016) and to some extent the limited literature on anxiety disorders (Reich & Braginsky, 1994; Taylor & Stopa, 2013). Taken together, these findings suggest a transdiagnostic role for IU, neuroticism and jumping to conclusions bias across SSDs and anxiety disorders, providing further evidence for shared negative affective temperament and cognitive biases across these disorders (Braga et al., 2013; Hall, 2018).

As expected, both paranoia and prodromal symptoms were highest in those with SSDs, then anxiety disorders and lowest in the non‐clinical group. The findings related to SSDs are in line with the extant literature, which suggests a central role of paranoia (e.g. Freeman, 2016) and prodromal symptoms (Yung et al., 1998, 2003) in SSDs. However, the findings related to those with anxiety disorders are relatively unique, given the limited literature on the presentation of paranoia (Reich & Braginsky, 1994; Taylor & Stopa, 2013) and prodromal symptoms (Rietdijk et al., 2011; Shioiri et al., 2007) in anxiety disorder populations. Such results support the notion that anxiety disorders may represent a precursor and risk factor for the development of SSDs (Hall, 2018).

The relationship between IU and paranoia/prodromal symptoms was transdiagnostic, as higher IU was associated with greater paranoia/prodromal symptoms across SSDs, anxiety disorders and a non‐clinical sample. Interestingly, the range of scores for IU and paranoia/prodromal symptoms was the largest for SSDs, then anxiety disorders and smallest for non‐clinical samples (see Figures 1 and 2), suggesting that these scores linearly increase in severity, potentially reflecting risk for psychosis. These findings align with a prior systematic review on IU and paranoia in community samples, at‐risk SSD samples and those with SSDs (Morriss, Butler, & Ellett, 2024). As far as we are aware, this is one of the first studies to demonstrate a relationship between IU and paranoia/prodromal symptoms in anxiety disorder populations.

Most of the relationships between IU and paranoia /prodromal symptoms across the different samples remained significant when controlling for neuroticism and the jumping to conclusions bias. However, the relationship between IU and paranoia in the SSD group was not significant over the jumping to conclusions bias. In general, these results suggest that IU has a unique relationship with paranoia/prodromal symptoms across these different populations, which highlights the potential of IU as a risk and/or maintaining factor of paranoia/prodromal symptoms. Notably, the relationship between IU and paranoia was not specific over the jumping to conclusions bias in SSDs. This result suggests that IU and the jumping to conclusions bias may be more closely aligned with paranoia in SSDs. Future research should examine the extent to which IU and jumping to conclusions bias may precede each other (e.g. sensing uncertainty may lead to jumping to a threatening conclusion) or possibly work together to elicit paranoid states.

The findings from this study have potential clinical implications for the development of new therapies that aim to target IU in SSDs and anxiety disorders. Previous research has demonstrated that gold‐standard treatments such as cognitive behavioural therapy (CBT) for anxiety disorders (Miller & McGuire, 2023) are effective in reducing IU and associated anxiety symptoms. The current findings raise the intriguing possibility that CBT for psychosis (Sitko et al., 2020) could also be modified to target IU in those experiencing paranoia/prodromal symptoms. However, this hypothesis needs to be tested in future intervention studies to determine whether reducing IU directly leads to a reduction in paranoia/prodromal symptoms and the prevention of psychotic episodes.

The study had several limitations. First, the study employed a cross‐sectional design, which prevents the establishment of causality. Second, the study relied entirely on self‐report measures. Further research using experimental and longitudinal designs should examine the extent to which IU may precede and maintain paranoia/prodromal symptoms, and whether altering IU leads to changes in paranoia/prodromal symptoms. Third, participants self‐reported their clinical diagnoses, who diagnosed them and whether they were currently receiving support from mental health services. This may limit the generalisability of our findings given that we recruited a convenience sample online. Future research might usefully employ more robust methods to establish clinical diagnoses, such as the use of diagnostic interviews or through the use of hospital records. Fourth, cronbach's alpha for the jumping to conclusions bias subscale was just below the acceptable level of 0.7, such that we should interpret these findings cautiously. Fifth, the groups varied by demographic factors such as gender, employment status and ethnicity. Therefore, further replication attempts should aim to match their groups on demographic variables. Finally, the sample is limited to those from English speaking countries. Future research should replicate these findings in diverse samples to fully understand generalisability.

Overall, this study suggests that the relationship between IU and paranoia/prodromal symptoms is transdiagnostic across SSD, anxiety disorders and non‐clinical samples. Moreover, these relationships are unique, over and above neuroticism and to some extent the jumping to conclusions bias. Further research using both experimental and longitudinal designs is required to determine how IU contributes to paranoia/prodromal symptoms, and whether targeting IU can alter paranoia/prodromal symptoms.

## AUTHOR CONTRIBUTIONS

**Jayne Morriss:** Visualization; writing – original draft; methodology; formal analysis. **Lyn Ellett:** Conceptualization; funding acquisition; methodology; writing – review and editing; project administration; data curation.

## CONFLICT OF INTEREST STATEMENT

The authors confirm that there are no conflicts of interest to declare.

## Data Availability

The data that support the findings of this study are available on request from the corresponding author. The data are not publicly available due to privacy or ethical restrictions.

## References

[papt12599-bib-0001] Barrantes‐Vidal, N. , Ros‐Morente, A. , & Kwapil, T. R. (2009). An examination of neuroticism as a moderating factor in the association of positive and negative schizotypy with psychopathology in a nonclinical sample. Schizophrenia Research, 115(2–3), 303–309. 19822406 10.1016/j.schres.2009.09.021

[papt12599-bib-0002] Berenbaum, H. , & Fujita, F. (1994). Schizophrenia and personality: Exploring the boundaries and connections between vulnerability and outcome. Journal of Abnormal Psychology, 103(1), 148–158. 8040476 10.1037//0021-843x.103.1.148

[papt12599-bib-0003] Braga, R. J. , Reynolds, G. P. , & Siris, S. G. (2013). Anxiety comorbidity in schizophrenia. Psychiatry Research, 210(1), 1–7. 23932838 10.1016/j.psychres.2013.07.030

[papt12599-bib-0004] Bredemeier, K. , McCole, K. , Luther, L. , Beck, A. T. , & Grant, P. M. (2019). Reliability and validity of a brief version of the intolerance of uncertainty scale in outpatients with psychosis. Journal of Psychopathology and Behavioral Assessment, 41, 221–234.

[papt12599-bib-0005] Broome, M. R. , Johns, L. C. , Valli, I. , Woolley, J. B. , Tabraham, P. , Brett, C. , & McGuire, P. K. (2007). Delusion formation and reasoning biases in those at clinical high risk for psychosis. The British Journal of Psychiatry, 191(S51), s38–s42. 10.1192/bjp.191.51.s38 18055936

[papt12599-bib-0006] Byrne, B. M. (2010). Structural equation modeling with AMOS: Basic concepts, applications, and programming. Routledge.

[papt12599-bib-0007] Carleton, R. N. (2016a). Fear of the unknown: One fear to rule them all? Journal of Anxiety Disorders, 41, 5–21. 27067453 10.1016/j.janxdis.2016.03.011

[papt12599-bib-0008] Carleton, R. N. (2016b). Into the unknown: A review and synthesis of contemporary models involving uncertainty. Journal of Anxiety Disorders, 39, 30–43. 26945765 10.1016/j.janxdis.2016.02.007

[papt12599-bib-0009] Carleton, R. N. , Mulvogue, M. K. , Thibodeau, M. A. , McCabe, R. E. , Antony, M. M. , & Asmundson, G. J. (2012). Increasingly certain about uncertainty: Intolerance of uncertainty across anxiety and depression. Journal of Anxiety Disorders, 26(3), 468–479. 22366534 10.1016/j.janxdis.2012.01.011

[papt12599-bib-0047] Carleton, R. N. , Norton, M. P. J. , & Asmundson, G. J. (2007). Fearing the unknown: A short version of the Intolerance of Uncertainty Scale. Journal of Anxiety Disorders, 21(1), 105–117. 16647833 10.1016/j.janxdis.2006.03.014

[papt12599-bib-0010] Clark, L. A. , Watson, D. , & Mineka, S. (1994). Temperament, personality, and the mood and anxiety disorders. Journal of Abnormal Psychology, 103(1), 103–116. 8040472

[papt12599-bib-0011] Cupid, J. , Stewart, K. E. , Sumantry, D. , & Koerner, N. (2021). Feeling safe: Judgements of safety and anxiety as a function of worry and intolerance of uncertainty. Behaviour Research and Therapy, 147, 103973. 34607250 10.1016/j.brat.2021.103973

[papt12599-bib-0012] Freeman, D. (2016). Persecutory delusions: A cognitive perspective on understanding and treatment. The Lancet Psychiatry, 3(7), 685–692. 27371990 10.1016/S2215-0366(16)00066-3

[papt12599-bib-0048] Freeman, D. , Loe, B. S. , Kingdon, D. , Startup, H. , Molodynski, A. , Rosebrock, L. , Brown, P. , Sheaves, B. , Waite, F. , & Bird, J. C. (2021). The revised Green et al., Paranoid Thoughts Scale (R‐GPTS): Psychometric properties, severity ranges, and clinical cut‐offs. Psychological Medicine, 51(2), 244–253. 31744588 10.1017/S0033291719003155 PMC7893506

[papt12599-bib-0013] Freeman, D. , Pugh, K. , & Garety, P. (2008). Jumping to conclusions and paranoid ideation in the general population. Schizophrenia Research, 102(1–3), 254–260. 18442898 10.1016/j.schres.2008.03.020

[papt12599-bib-0014] Freeman, D. , Startup, H. , Dunn, G. , Černis, E. , Wingham, G. , Pugh, K. , Cordwell, J. , & Kingdon, D. (2013). The interaction of affective with psychotic processes: A test of the effects of worrying on working memory, jumping to conclusions, and anomalies of experience in patients with persecutory delusions. Journal of Psychiatric Research, 47(12), 1837–1842. 23871449 10.1016/j.jpsychires.2013.06.016 PMC3905189

[papt12599-bib-0015] Freeston, M. H. , Rhéaume, J. , Letarte, H. , Dugas, M. J. , & Ladouceur, R. (1994). Why do people worry? Personality and Individual Differences, 17(6), 791–802.

[papt12599-bib-0016] Hall, J. (2018). Schizophrenia—An anxiety disorder? British Journal of Psychiatry, 211(5), 262–263. 10.1192/bjp.bp.116.195370 29092833

[papt12599-bib-0017] Hong, R. Y. , & Lee, S. S. (2015). Further clarifying prospective and inhibitory intolerance of uncertainty: Factorial and construct validity of test scores from the intolerance of uncertainty scale. Psychological Assessment, 27(2), 605. 25602690 10.1037/pas0000074

[papt12599-bib-0018] Ising, H. K. , Veling, W. , Loewy, R. L. , Rietveld, M. W. , Rietdijk, J. , Dragt, S. , Klaassen, R. M. , Nieman, D. H. , Wunderink, L. , Linszen, D. H. , & van der Gaag, M. (2012). The validity of the 16‐item version of the prodromal questionnaire (PQ‐16) to screen for ultra high risk of developing psychosis in the general help‐seeking population. Schizophrenia Bulletin, 38(6), 1288–1296. 22516147 10.1093/schbul/sbs068 PMC3713086

[papt12599-bib-0019] John, O. P. (2021). History, measurement, and conceptual elaboration of the big‐five trait taxonomy: The paradigm matures. In O. P. John & R. W. Robins (Eds.), Handbook of personality: Theory and research (4th ed., pp. 35–82). The Guilford Press.

[papt12599-bib-0020] Krabbendam, L. , Janssen, I. , Bak, M. , Bijl, R. V. , de Graaf, R. , & van Os, J. (2002). Neuroticism and low self‐esteem as risk factors for psychosis. Social Psychiatry and Psychiatric Epidemiology, 37, 1–6. 11924745 10.1007/s127-002-8207-y

[papt12599-bib-0021] Lebert, L. , Turkington, D. , Freeston, M. , & Dudley, R. (2021). Rumination, intolerance of uncertainty and paranoia in treatment resistant psychosis. Psychosis, 13(1), 65–70.

[papt12599-bib-0022] Li, J. , Gao, J. , Zhang, Q. , Li, C. , & Cui, L. (2021). The efficacy of intolerance of uncertainty intervention on anxiety and its mediating role by multilayer linear model analysis. Journal of Psychopathology and Behavioral Assessment, 43, 142–151.

[papt12599-bib-0023] Lopes, B. C. , & Jaspal, R. (2025). Exposure to ghosting, Gaslighting, and coercion and mental health outcomes. Partner Abuse.

[papt12599-bib-0024] Mahoney, A. E. , & McEvoy, P. M. (2012). Trait versus situation‐specific intolerance of uncertainty in a clinical sample with anxiety and depressive disorders. Cognitive Behaviour Therapy, 41(1), 26–39. 22032804 10.1080/16506073.2011.622131

[papt12599-bib-0025] McEvoy, P. M. , Hyett, M. P. , Shihata, S. , Price, J. E. , & Strachan, L. (2019). The impact of methodological and measurement factors on transdiagnostic associations with intolerance of uncertainty: A meta‐analysis. Clinical Psychology Review, 73, 101778. 31678816 10.1016/j.cpr.2019.101778

[papt12599-bib-0026] Miller, M. L. , & McGuire, J. F. (2023). Targeting intolerance of uncertainty in treatment: A meta‐analysis of therapeutic effects, treatment moderators, and underlying mechanisms. Journal of Affective Disorders, 341, 283–295. 37657623 10.1016/j.jad.2023.08.132

[papt12599-bib-0027] Morriss, J. , Abend, R. , Zika, O. , Bradford, D. E. , & Mertens, G. (2023). Neural and psychophysiological markers of intolerance of uncertainty. International Journal of Psychophysiology, 184, 94–99. 36630825 10.1016/j.ijpsycho.2023.01.003

[papt12599-bib-0028] Morriss, J. , Butler, D. , & Ellett, L. (2024). Intolerance of uncertainty and psychosis: A systematic review. British Journal of Clinical Psychology, 64(2), 344–354. 39438423 10.1111/bjc.12509 PMC12057307

[papt12599-bib-0029] Morriss, J. , Gaudiano, B. A. , So, S. H. , Kingston, J. , Lincoln, T. , Morris, E. M. , & Ellett, L. (2024). Associations between intolerance of uncertainty, paranoia, anxiety, and depression: Evidence from an international multisite sample. Mental Health Science, 1, e81.

[papt12599-bib-0030] Morriss, J. , Goh, K. , Hirsch, C. R. , & Dodd, H. F. (2023). Intolerance of uncertainty heightens negative emotional states and dampens positive emotional states. Frontiers in Psychiatry, 14, 1147970. 37032949 10.3389/fpsyt.2023.1147970 PMC10073686

[papt12599-bib-0031] Pepperdine, E. , Lomax, C. , & Freeston, M. (2018). Disentangling intolerance of uncertainty and threat appraisal in everyday situations. Journal of Anxiety Disorders, 57, 21–38. 10.1016/j.janxdis.2018.04.002 29724665

[papt12599-bib-0032] Peters, E. R. , Moritz, S. , Schwannauer, M. , Wiseman, Z. , Greenwood, K. E. , Scott, J. , Beck, A. T. , Donaldson, C. , Hagen, R. , Ross, K. , Veckenstedt, R. , Ison, R. , Williams, S. , Kuipers, E. , & Garety, P. A. (2014). Cognitive biases questionnaire for psychosis. Schizophrenia Bulletin, 40(2), 300–313. 23413104 10.1093/schbul/sbs199 PMC3932080

[papt12599-bib-0033] Rammstedt, B. , & John, O. P. (2007). Measuring personality in one minute or less: A 10‐item short version of the big five inventory in English and German. Journal of Research in Personality, 41(1), 203–212.

[papt12599-bib-0034] Reich, J. , & Braginsky, Y. (1994). Paranoid personality traits in a panic disorder population: A pilot study. Comprehensive Psychiatry, 35(4), 260–264. 7956181 10.1016/0010-440x(94)90017-5

[papt12599-bib-0035] Rietdijk, J. , Hogerzeil, S. J. , van Hemert, A. M. , Cuijpers, P. , Linszen, D. H. , & van der Gaag, M. (2011). Pathways to psychosis: Help‐seeking behavior in the prodromal phase. Schizophrenia Research, 132(2–3), 213–219. 21907547 10.1016/j.schres.2011.08.009

[papt12599-bib-0036] Shihata, S. , McEvoy, P. M. , Mullan, B. A. , & Carleton, R. N. (2016). Intolerance of uncertainty in emotional disorders: What uncertainties remain? Journal of Anxiety Disorders, 41, 115–124. 27212227 10.1016/j.janxdis.2016.05.001

[papt12599-bib-0037] Shioiri, T. , Shinada, K. , Kuwabara, H. , & Someya, T. (2007). Early prodromal symptoms and diagnoses before first psychotic episode in 219 inpatients with schizophrenia. Psychiatry and Clinical Neurosciences, 61(4), 348–354. 17610658 10.1111/j.1440-1819.2007.01685.x

[papt12599-bib-0038] Sitko, K. , Bewick, B. M. , Owens, D. , & Masterson, C. (2020). Meta‐analysis and meta‐regression of cognitive behavioral therapy for psychosis (CBTp) across time: The effectiveness of CBTp has improved for delusions. Schizophrenia Bulletin Open, 1(1), sgaa023.

[papt12599-bib-0039] So, S. H. W. , Siu, N. Y. F. , Wong, H. L. , Chan, W. , & Garety, P. A. (2016). ‘Jumping to conclusions’ data‐gathering bias in psychosis and other psychiatric disorders—Two meta‐analyses of comparisons between patients and healthy individuals. Clinical Psychology Review, 46, 151–167. 27216559 10.1016/j.cpr.2016.05.001

[papt12599-bib-0040] Tanovic, E. , Gee, D. G. , & Joormann, J. (2018). Intolerance of uncertainty: Neural and psychophysiological correlates of the perception of uncertainty as threatening. Clinical Psychology Review, 60, 87–99. 29331446 10.1016/j.cpr.2018.01.001

[papt12599-bib-0041] Taylor, K. N. , & Stopa, L. (2013). The fear of others: A pilot study of social anxiety processes in paranoia. Behavioural and Cognitive Psychotherapy, 41(1), 66–88. 23017737 10.1017/S1352465812000690

[papt12599-bib-0043] White, R. G. , & Gumley, A. (2010). Intolerance of uncertainty and distress associated with the experience of psychosis. Psychology and Psychotherapy: Theory, Research and Practice, 83(3), 317–324. 10.1348/147608309X477572 19917154

[papt12599-bib-0044] Yung, A. R. , & McGorry, P. D. (1996). The prodromal phase of first‐episode psychosis: Past and current conceptualizations. Schizophrenia Bulletin, 22(2), 353–370. 8782291 10.1093/schbul/22.2.353

[papt12599-bib-0045] Yung, A. R. , Phillips, L. J. , McGorry, P. D. , McFarlane, C. A. , Francey, S. , Harrigan, S. , & Jackson, H. J. (1998). Prediction of psychosis: A step towards indicated prevention of schizophrenia. The British Journal of Psychiatry, 172(S33), 14–20. 9764121

[papt12599-bib-0046] Yung, A. R. , Phillips, L. J. , Yuen, H. P. , Francey, S. M. , McFarlane, C. A. , Hallgren, M. , & McGorry, P. D. (2003). Psychosis prediction: 12‐month follow up of a high‐risk (“prodromal”) group. Schizophrenia Research, 60(1), 21–32. 12505135 10.1016/s0920-9964(02)00167-6

